# Supplementary figures and images for: Identifying high risk clinical phenogroups of pulmonary hypertension through a clustering analysis
Source: PLoS One. 2023 Aug 25;18(8):e0290553. doi: 10.1371/journal.pone.0290553 (PMC10456132; doi:10.1371/journal.pone.0290553)

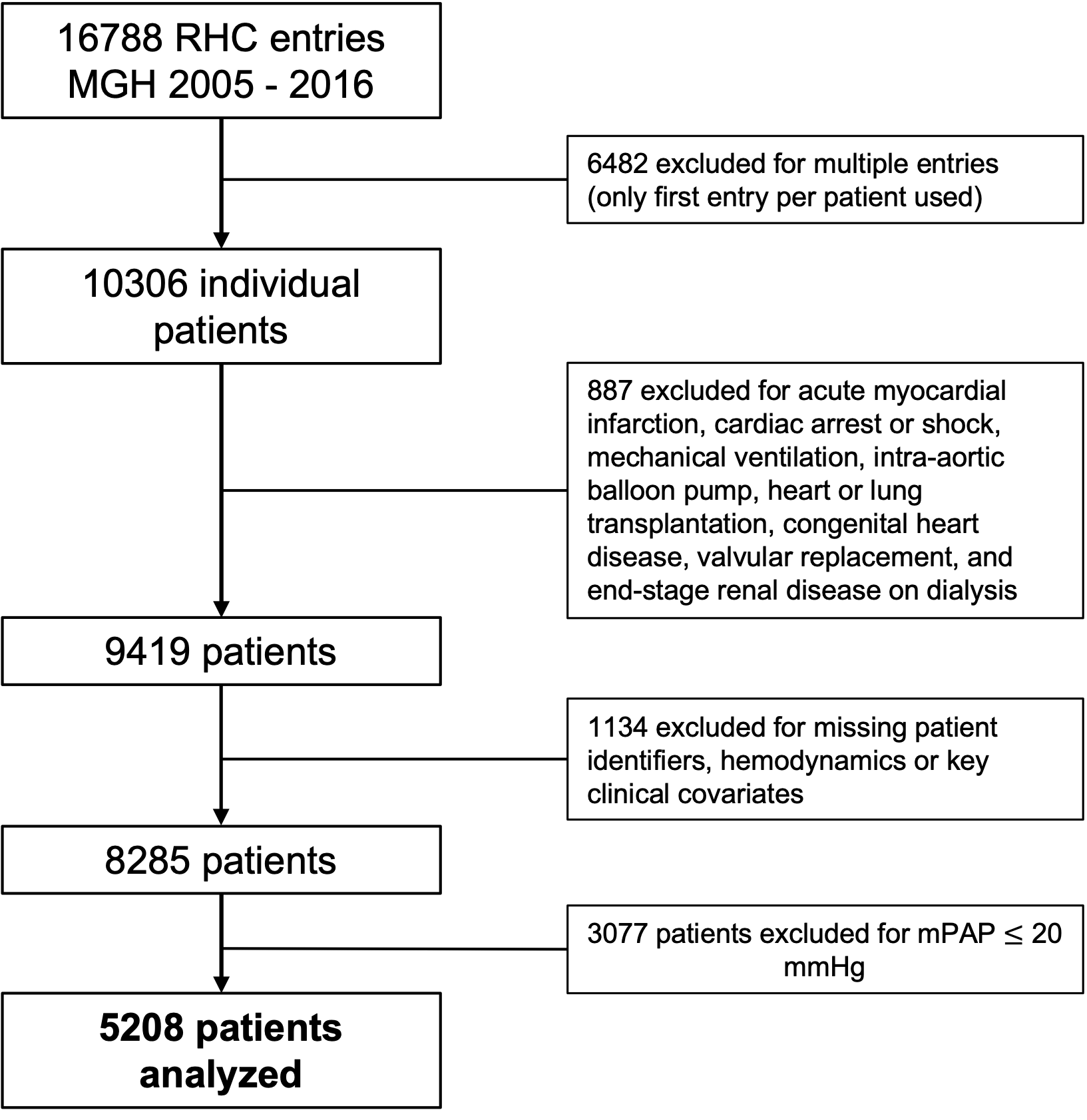

Supplement: S1 Fig — (TIFF) [file pone.0290553.s001.tiff]
