# Supplementary material for: Identifying high risk clinical phenogroups of pulmonary hypertension through a clustering analysis
Source: PLoS One. 2023 Aug 25;18(8):e0290553. doi: 10.1371/journal.pone.0290553 (PMC10456132; doi:10.1371/journal.pone.0290553)
Supplement: S1 Table — (PDF) [file pone.0290553.s002.pdf]

Supplemental Table 1A: Heart Failure Hospitalization		
**Code Type**	**Code**	**Description**
ICD-9 (Heart Failure)	398.91, 402.01, 402.11, 402.91, 404.01, 404.03, 404.11, 404.13, 404.91, 404.93, 428, 428.1, 428.2, 428.21, 428.22, 428.23, 428.3, 428.31, 428.32, 428.33, 428.4, 428.41, 428.42, 428.43, 428.9	Rheumatic heart failure (congestive), Malignant hypertensive heart disease with heart failure, Benign hypertensive heart disease with heart failure, Unspecified hypertensive heart disease with heart failure , Hypertensive heart and chronic kidney disease, malignant, with heart failure and with chronic kidney disease stage I through stage IV, or unspecified, Hypertensive heart and chronic kidney disease, malignant, with heart failure and with chronic kidney disease stage V or end stage renal disease, Hypertensive heart and chronic kidney disease, benign, with heart failure and with chronic kidney disease stage I through stage IV, or unspecified, Hypertensive heart and chronic kidney disease, benign, with heart failure and chronic kidney disease stage V or end stage renal disease, Hypertensive heart and chronic kidney disease, unspecified, with heart failure and with chronic kidney disease stage I through stage IV, or unspecified, Hypertensive heart and chronic kidney disease, unspecified, with heart failure and chronic kidney disease stage V or end stage renal disease, Congestive heart failure, unspecified, Left heart failure, Systolic heart failure, unspecified, Acute systolic heart failure, Chronic systolic heart failure, Acute on chronic systolic heart failure, Diastolic heart failure, unspecified, Acute diastolic heart failure, Chronic diastolic heart failure, Acute on chronic diastolic heart failure, Combined systolic and diastolic heart failure, unspecified, Acute combined systolic and diastolic heart failure, Chronic combined systolic and diastolic heart failure, Acute on chronic combined systolic and diastolic heart failure, Heart failure, unspecified
ICD-10 (Heart Failure)	I09.81, I11.0, I13.0, I13.2, I50.1, I50.20, I50.21, I50.22, I50.23, I50.30, I50.31, I50.32, I50.33, I50.40, I50.41, I50.42, I50.43, I50.9, I97.130, I97.131	Rheumatic Heart Failure, Hypertensive Heart Disease With Heart Failure, Hypertensive Heart And Chronic Kidney Disease With Heart Failure And Stage 1 Through Stage 4 Chronic Kidney Disease, Or Unspecified Chronic Kidney Disease, Hypertensive Heart And Chronic Kidney Disease With Heart Failure And With Stage 5 Chronic Kidney Disease, Or End Stage Renal Disease, Left Ventricular Failure, Unspecified Systolic (Congestive) Heart Failure, Acute Systolic (Congestive) Heart Failure, Chronic Systolic (Congestive) Heart Failure, Acute On Chronic Systolic (Congestive) Heart Failure, Unspecified Diastolic (Congestive) Heart Failure, Acute Diastolic (Congestive) Heart Failure, Chronic Diastolic (Congestive) Heart Failure, Acute On Chronic Diastolic (Congestive) Heart Failure, Unspecified Combined Systolic (Congestive) And Diastolic (Congestive) Heart Failure, Acute Combined Systolic (Congestive) And Diastolic (Congestive) Heart Failure, Chronic Combined Systolic (Congestive) And Diastolic (Congestive) Heart Failure, Acute On Chronic Combined Systolic (Congestive) And Diastolic (Congestive) Heart Failure, Heart Failure, Unspecified, Postprocedural heart failure following cardiac surgery, Postprocedural heart failure following other surgery
CPT (Ventricular Assist Device)	33990, 33991, 33975, 33976, 33979	Insert ventricular assist device, percutaneous, arterial access only; insert VAD, percutaneous, arterial & venous access, transseptal; insertion of ventricular assist device , extracorporeal, single ventricle; insertion of ventricular assist device, extracorporeal, biventricular; insertion of ventricular assist device, implantable, intracorporeal, single ventricle

CPT (Orthotopic Heart Transplant)	33933, 33935, 33944, 33945	33933 and 33935 are heart-lung; 33944 and 33945 are heart-only transplant

**Supplemental Table 1B: Acute Myocardial Infarction Hospitalization**		
**Code Type**	**Code**	**Description**
ICD-9 (Acute myocardial infarction)	410, 410.01, 410.02, 410.1, 410.11, 410.12, 410.2, 410.21, 410.22, 410.3, 410.31, 410.32, 410.4, 410.41, 410.42, 410.5, 410.51, 410.52, 410.6, 410.61, 410.62, 410.7, 410.71, 410.72, 410.8, 410.81, 410.82, 410.9, 410.91, 410.92, 412, 429.79	Acute myocardial infarction of anterolateral wall, episode of care unspecified, Acute myocardial infarction of anterolateral wall, initial episode of care, Acute myocardial infarction of anterolateral wall, subsequent episode of care , Acute myocardial infarction of other anterior wall, episode of care unspecified, Acute myocardial infarction of other anterior wall, initial episode of care, Acute myocardial infarction of other anterior wall, subsequent episode of care, Acute myocardial infarction of inferolateral wall, episode of care unspecified, Acute myocardial infarction of inferolateral wall, initial episode of care, Acute myocardial infarction of inferolateral wall, subsequent episode of care, Acute myocardial infarction of inferoposterior wall, episode of care unspecified, Acute myocardial infarction of inferoposterior wall, initial episode of care, Acute myocardial infarction of inferoposterior wall, subsequent episode of care, Acute myocardial infarction of other inferior wall, episode of care unspecified, Acute myocardial infarction of other inferior wall, initial episode of care, Acute myocardial infarction of other inferior wall, subsequent episode of care, Acute myocardial infarction of other lateral wall, episode of care unspecified, Acute myocardial infarction of other lateral wall, initial episode of care, Acute myocardial infarction of other lateral wall, subsequent episode of care, True posterior wall infarction, episode of care unspecified, True posterior wall infarction, initial episode of care, True posterior wall infarction, subsequent episode of care, Subendocardial infarction, episode of care unspecified, Subendocardial infarction, initial episode of care, Subendocardial infarction, subsequent episode of care, Acute myocardial infarction of other specified sites, episode of care unspecified, Acute myocardial infarction of other specified sites, initial episode of care, Acute myocardial infarction of other specified sites, subsequent episode of care, Acute myocardial infarction of unspecified site, episode of care unspecified, Acute myocardial infarction of unspecified site, initial episode of care, Acute myocardial infarction of unspecified site, subsequent episode of care, Old myocardial infarction , Certain sequelae of myocardial infarction, not elsewhere classified, other
ICD-10 (acute myocardial infraction)	I21.01, I21.02, I21.09, I21.11, I21.19, I21.21, I21.29, I21.3, I21.4, I22.0, I22.1, I22.2, I22.8, I22.9, I23.0, I23.1, I23.2, I23.3, I23.4, I23.5, I23.6, I23.7, I23.8, I24.1, I25.2	ST elevation (STEMI) myocardial infarction involving left main coronary artery, ST elevation (STEMI) myocardial infarction involving left anterior descending coronary artery, ST elevation (STEMI) myocardial infarction involving other coronary artery of anterior wall, ST elevation (STEMI) myocardial infarction involving right coronary artery, ST elevation (STEMI) myocardial infarction involving other coronary artery of inferior wall, ST elevation (STEMI) myocardial infarction involving left circumflex coronary artery, ST elevation (STEMI) myocardial infarction involving other sites, ST elevation (STEMI) myocardial infarction of unspecified site , Non-ST elevation (NSTEMI) myocardial infarction , Subsequent ST elevation (STEMI) myocardial infarction of anterior wall, Subsequent ST

		elevation (STEMI) myocardial infarction of inferior wall, Subsequent non-ST elevation (NSTEMI) myocardial infarction , Subsequent ST elevation (STEMI) myocardial infarction of other sites, Subsequent ST elevation (STEMI) myocardial infarction of unspecified site, Hemopericardium as current complication following acute myocardial infarction, Atrial septal defect as current complication following acute myocardial infarction, Ventricular septal defect as current complication following acute myocardial infarction, Rupture of cardiac wall without hemopericardium as current complication following acute myocardial infarction, Rupture of chordae tendineae as current complication following acute myocardial infarction, Rupture of papillary muscle as current complication following acute myocardial infarction, Thrombosis of atrium, auricular appendage, and ventricle as current complications following acute myocardial infarction, Postinfarction angina, Other current complications following acute myocardial infarction, Dressler's syndrome, Old myocardial infarction
CPT	33510, 33511, 33512, 33513, 33514, 33516, 33517, 33518, 33519, 33521, 33522, 33523, 33533, 33534, 33535, 33536	coronary artery bypass graft
CPT	92920, 92921, 92924, 92925, 92928, 92929, 92933, 92934, 92937, 92938, **92941**, 92943, 92944, 92973	percutaneous coronary intervention

**Supplemental Table 1C: Stroke or Transient Ischemic Attack**		
**Code Type**	**Code**	**Description**
ICD-9	362.31, 362.32, 362.33, 362.34, 388.02, 430, 431, 432.9, 433.01, 433.11, 433.21, 433.31, 433.81, 433.91, 434, 434.01, 434.1, 434.11, 434.9, 434.91, 435, 435.1, 435.2, 435.3, 435.8, 435.9, 437.1, 437.7, 437.9, 438.1, 438.11, 438.12, 438.13, 438.14, 438.2, 438.21, 438.22, 438.81, 438.82, 438.83, 438.89, 438.9, 997.02, V12.54	Central retinal artery occlusion, Retinal arterial branch occlusion, Partial retinal arterial occlusion, Transient retinal arterial occlusion, Transient ischemic deafness, Subarachnoid hemorrhage, Intracerebral hemorrhage, Unspecified intracranial hemorrhage, Occlusion and stenosis of basilar artery with cerebral infarction, Occlusion and stenosis of carotid artery with cerebral infarction, Occlusion and stenosis of vertebral artery with cerebral infarction, Occlusion and stenosis of multiple and bilateral precerebral arteries with cerebral infarction, Occlusion and stenosis of other specified precerebral artery with cerebral infarction, Occlusion and stenosis of unspecified precerebral artery with cerebral infarction, Cerebral thrombosis without mention of cerebral infarction, Cerebral thrombosis with cerebral infarction, Cerebral embolism without mention of cerebral infarction, Cerebral embolism with cerebral infarction, Cerebral artery occlusion, unspecified without mention of cerebral infarction, Cerebral artery occlusion, unspecified with cerebral infarction, Basilar artery syndrome, Vertebral artery syndrome , Subclavian steal syndrome, Vertebrobasilar artery syndrome, Other specified transient cerebral ischemia, Unspecified

		transient cerebral ischemia, Other generalized ischemic cerebrovascular disease, Transient global amnesia, Unspecified cerebrovascular disease, Late effects of cerebrovascular disease, speech and language deficit, unspecified, Late effects of cerebrovascular disease, aphasia, Late effects of cerebrovascular disease, dysphasia, Late effects of cerebrovascular disease, dysarthria, Late effects of cerebrovascular disease, fluency disorder, Late effects of cerebrovascular disease, hemiplegia affecting unspecified side, Late effects of cerebrovascular disease, hemiplegia affecting dominant side, Late effects of cerebrovascular disease, hemiplegia affecting nondominant side, Other late effects of cerebrovascular disease, apraxia, Other late effects of cerebrovascular disease, dysphagia, Other late effects of cerebrovascular disease, facial weakness, Other late effects of cerebrovascular disease, Unspecified late effects of cerebrovascular disease, Iatrogenic cerebrovascular infarction or hemorrhage, Personal history of transient ischemic attack (TIA), and cerebral infarction without residual deficits
ICD-10	G45.0, G45.1, G45.2, G45.3, G45.4, G45.8, G46.3, G46.4, H34.00, H34.01, H34.02, H34.03, H34.10, H34.11, H34.12, H34.13, H34.211, H34.212, H34.213, H34.219, H34.231, H34.232, H34.233, H34.239, H93.099, I60.9, I61.9, I62.9, I63.00, I63.011, I63.012, I63.019, I63.111, I63.112, I63.119, I63.12, I63.131, I63.132, I63.139, I63.19, I63.20, I63.211, I63.212, I63.219, I63.22, I63.231, I63.232, I63.239, I63.29, I63.30, I63.311, I63.312, I63.319, I63.321, I63.322, I63.329, I63.331, I63.332, I63.339, I63.341, I63.342, I63.349, I63.40, I63.411, I63.412, I63.419, I63.421, I63.422, I63.429, I63.431, I63.432, I63.439, I63.49, I63.50, I63.511, I63.512, I63.519,	Vertebro-Basilar Artery Syndrome, Carotid Artery Syndrome, Multiple and bilateral precerebral artery syndromes, Amaurosis fugax, Transient Global Amnesia, Other transient cerebral ischemic attacks and related syndromes, Brain stem stroke syndrome, Cerebellar stroke syndrome, Transient Retinal Artery Occlusion, Unspecified Eye, Transient retinal artery occlusion, right eye, Transient retinal artery occlusion, left eye, Transient retinal artery occlusion, bilateral, Central retinal artery occlusion, unspecified eye, Central retinal artery occlusion, right eye, Central retinal artery occlusion, left eye, Central retinal artery occlusion, bilateral, Partial retinal artery occlusion, right eye, Partial retinal artery occlusion, left eye, Partial retinal artery occlusion, bilateral, Partial Retinal Artery Occlusion, Unspecified Eye, Retinal artery branch occlusion, right eye, Retinal artery branch occlusion, left eye, Retinal artery branch occlusion, bilateral, Retinal Artery Branch Occlusion, Unspecified Eye, Unspecified Degenerative and Vascular Disorders of Unspecified Ear, Nontraumatic Subarachnoid Hemorrhage, Unspecified, Nontraumatic intracerebral Hemorrhage, unspecified, Nontraumatic Intracranial Hemorrhage, Unspecified, Cerebral infarction due to thrombosis of unspecified precerebral artery, Cerebral infarction due to thrombosis of right vertebral artery, Cerebral infarction due to thrombosis of left vertebral artery, Cerebral infarction due to thrombosis of unspecified vertebral artery, Cerebral infarction due to embolism of right vertebral artery, Cerebral infarction due to embolism of left vertebral artery, Cerebral infarction due to embolism of unspecified vertebral artery , Cerebral infarction due to embolism of basilar artery, Cerebral infarction due to embolism of right carotid artery, Cerebral infarction due to embolism of left carotid artery, Cerebral infarction due to embolism of unspecified carotid artery, Cerebral infarction due to embolism of other precerebral artery, Cerebral infarction due to unspecified occlusion or stenosis of unspecified precerebral arteries , Cerebral infarction due to unspecified occlusion or stenosis of right vertebral arteries, Cerebral infarction due to unspecified occlusion or stenosis of left vertebral arteries, Cerebral infarction due to unspecified occlusion or stenosis of unspecified vertebral arteries, Cerebral Infraction Due to Unspecified Occlusion or Stenosis of Basilar Arteries, Cerebral infarction due to unspecified occlusion or stenosis of right carotid arteries, Cerebral infarction due to unspecified

	I63.521, I63.522, I63.529, I63.531, I63.532, I63.539, I63.541, I63.542, I63.549, I63.59, I63.6, I63.8, I63.9, I66.01, I66.02, I66.03, I66.09, I66.11, I66.12, I66.13, I66.19, I66.21, I66.22, I66.23, I66.29, I66.3, I66.8, I66.9, I67.81, I67.82, I67.841, I67.848, I67.89, I67.9, I69.80, I69.81, I69.820, I69.821, I69.822, I69.823, I69.828, I69.831, I69.832, I69.833, I69.834, I69.839, I69.841, I69.842, I69.843, I69.844, I69.849, I69.851, I69.852, I69.853, I69.854, I69.859, I69.861, I69.862, I69.863, I69.864, I69.865, I69.869, I69.890, I69.891, I69.892, I69.893, I69.898, I69.90, I69.91, I69.920, I69.921, I69.922, I69.923, I69.928, I69.931, I69.932, I69.933, I69.934, I69.939, I69.941, I69.942, I69.943, I69.944, I69.949, I69.951, I69.952, I69.953, I69.954, I69.959, I69.961, I69.962, I69.963, I69.964, I69.965, I69.969, I69.990, I69.991, I69.992, I69.993, I69.998, I97.810, I97.811, I97.820, I97.821,	occlusion or stenosis of left carotid arteries, Cerebral infarction due to unspecified occlusion or stenosis of unspecified carotid arteries, Cerebral infarction due to unspecified occlusion or stenosis of other precerebral arteries, Cerebral infarction due to thrombosis of unspecified cerebral artery , Cerebral infarction due to thrombosis of right middle cerebral artery, Cerebral infarction due to thrombosis of left middle cerebral artery, Cerebral infarction due to thrombosis of unspecified middle cerebral artery, Cerebral infarction due to thrombosis of right anterior cerebral artery, Cerebral infarction due to thrombosis of left anterior cerebral artery, Cerebral infarction due to thrombosis of unspecified anterior cerebral artery, Cerebral infarction due to thrombosis of right posterior cerebral artery, Cerebral infarction due to thrombosis of left posterior cerebral artery, Cerebral infarction due to thrombosis of unspecified posterior cerebral artery, Cerebral infarction due to thrombosis of right cerebellar artery, Cerebral infarction due to thrombosis of left cerebellar artery, Cerebral infarction due to thrombosis of unspecified cerebellar artery, Cerebral infarction due to embolism of unspecified cerebral artery , Cerebral infarction due to embolism of right middle cerebral artery, Cerebral infarction due to embolism of left middle cerebral artery, Cerebral infarction due to embolism of unspecified middle cerebral artery, Cerebral infarction due to embolism of right anterior cerebral artery, Cerebral infarction due to embolism of left anterior cerebral artery, Cerebral infarction due to embolism of unspecified anterior cerebral artery, Cerebral infarction due to embolism of right posterior cerebral artery, Cerebral infarction due to embolism of left posterior cerebral artery, Cerebral infarction due to embolism of unspecified posterior cerebral artery , Cerebral infarction due to embolism of other cerebral artery, Cerebral infarction due to unspecified occlusion or stenosis of unspecified cerebral artery , Cerebral infarction due to unspecified occlusion or stenosis of right middle cerebral artery, Cerebral infarction due to unspecified occlusion or stenosis of left middle cerebral artery, Cerebral infarction due to unspecified occlusion or stenosis of unspecified middle cerebral artery, Cerebral infarction due to unspecified occlusion or stenosis of right anterior cerebral artery, Cerebral infarction due to unspecified occlusion or stenosis of left anterior cerebral artery, Cerebral infarction due to unspecified occlusion or stenosis of unspecified anterior cerebral artery, Cerebral infarction due to unspecified occlusion or stenosis of right posterior cerebral artery, Cerebral infarction due to unspecified occlusion or stenosis of left posterior cerebral artery, Cerebral infarction due to unspecified occlusion or stenosis of unspecified posterior cerebral artery, Cerebral infarction due to unspecified occlusion or stenosis of right cerebellar artery, Cerebral infarction due to unspecified occlusion or stenosis of left cerebellar artery, Cerebral infarction due to unspecified occlusion or stenosis of unspecified cerebellar artery, Cerebral infarction due to unspecified occlusion or stenosis of other cerebral artery , Cerebral infarction due to cerebral venous thrombosis, nonpyrogenic, Other cerebral infarction, Cerebral infarction, unspecified, Occlusion and stenosis of right middle cerebral artery, Occlusion and stenosis of left middle cerebral artery, Occlusion and stenosis of bilateral middle cerebral arteries, Occlusion and stenosis of unspecified middle cerebral artery, Occlusion and stenosis of right anterior cerebral artery, Occlusion and stenosis of left anterior cerebral artery, Occlusion and stenosis of bilateral anterior cerebral arteries, Occlusion and stenosis of unspecified

		anterior cerebral artery, Occlusion and stenosis of right posterior cerebral artery, Occlusion and stenosis of left posterior cerebral artery, Occlusion and stenosis of bilateral posterior cerebral arteries, Occlusion and stenosis of unspecified posterior cerebral artery , Occlusion and stenosis of cerebellar arteries, Occlusion and stenosis of other cerebral arteries, Occlusion and stenosis of unspecified cerebral artery, Acute cerebrovascular insufficiency, Cerebral ischemia, Reversible cerebrovascular vasoconstriction syndrome, Other cerebrovascular vasospasm and vasoconstriction , Other cerebrovascular disease, Cerebrovascular Disease, unspecified, Unspecified sequelae of other cerebrovascular disease, Cognitive deficits following other cerebrovascular disease, Aphasia following other cerebrovascular disease, Dysphasia following other cerebrovascular disease, Dysarthria following other cerebrovascular disease, Fluency disorder following other cerebrovascular disease, Other speech and language deficits following other cerebrovascular disease, Monoplegia of upper limb following other cerebrovascular disease affecting right dominant side, Monoplegia of upper limb following other cerebrovascular disease affecting left dominant side, Monoplegia of upper limb following other cerebrovascular disease affecting right non-dominant side, Monoplegia of upper limb following other cerebrovascular disease affecting left non-dominant side, Monoplegia of upper limb following other cerebrovascular disease affecting unspecified side, Monoplegia of lower limb following other cerebrovascular disease affecting right dominant side, Monoplegia of lower limb following other cerebrovascular disease affecting left dominant side, Monoplegia of lower limb following other cerebrovascular disease affecting right non-dominant side, Monoplegia of lower limb following other cerebrovascular disease affecting left non-dominant side, Monoplegia of lower limb following other cerebrovascular disease affecting unspecified side, Hemiplegia and hemiparesis following other cerebrovascular disease affecting right dominant side, Hemiplegia and hemiparesis following other cerebrovascular disease affecting left dominant side, Hemiplegia and hemiparesis following other cerebrovascular disease affecting right non-dominant side, Hemiplegia and hemiparesis following other cerebrovascular disease affecting left non-dominant side, Hemiplegia and hemiparesis following other cerebrovascular disease affecting unspecified side, Other paralytic syndrome following other cerebrovascular disease affecting right dominant side, Other paralytic syndrome following other cerebrovascular disease affecting left dominant side, Other paralytic syndrome following other cerebrovascular disease affecting right non-dominant side, Other paralytic syndrome following other cerebrovascular disease affecting left non-dominant side, Other paralytic syndrome following other cerebrovascular disease, bilateral, Other paralytic syndrome following other cerebrovascular disease affecting unspecified side, Apraxia following other cerebrovascular disease, Dysphagia following other cerebrovascular disease, Facial weakness following other cerebrovascular disease, Ataxia following other cerebrovascular disease, Other sequelae of other cerebrovascular disease , Unspecified Sequelae of unspecified cerebrovascular disease, Cognitive deficits following unspecified cerebrovascular disease, Aphasia following unspecified cerebrovascular disease, Dysphasia following unspecified cerebrovascular disease , Dysarthria following unspecified Cerebrovascular disease, Fluency disorder following

		unspecified cerebrovascular disease , Other speech and language deficits following unspecified cerebrovascular disease, Monoplegia of upper limb following unspecified cerebrovascular disease affecting right dominant side, Monoplegia of upper limb following unspecified cerebrovascular disease affecting left dominant side, Monoplegia of upper limb following unspecified cerebrovascular disease affecting right non-dominant side, Monoplegia of upper limb following unspecified cerebrovascular disease affecting left non-dominant side, Monoplegia of upper limb following unspecified cerebrovascular disease affecting unspecified side, Monoplegia of lower limb following unspecified cerebrovascular disease affecting right dominant side, Monoplegia of lower limb following unspecified cerebrovascular disease affecting left dominant side, Monoplegia of lower limb following unspecified cerebrovascular disease affecting right non-dominant side, Monoplegia of lower limb following unspecified cerebrovascular disease affecting left non-dominant side, Monoplegia of lower limb following unspecified cerebrovascular disease affecting unspecified side, Hemiplegia and hemiparesis following unspecified cerebrovascular disease affecting right dominant side , Hemiplegia and hemiparesis following unspecified cerebrovascular disease affecting left dominant side , Hemiplegia and hemiparesis following unspecified cerebrovascular disease affecting right non-dominant side, Hemiplegia and hemiparesis following unspecified cerebrovascular disease affecting left non-dominant side , Hemiplegia and hemiparesis following unspecified cerebrovascular disease affecting unspecified side , Other paralytic syndrome following unspecified cerebrovascular disease affecting right dominant side, Other paralytic syndrome following unspecified cerebrovascular disease affecting left dominant side, Other paralytic syndrome following unspecified cerebrovascular disease affecting right non-dominant side, Other paralytic syndrome following unspecified cerebrovascular disease affecting left non-dominant side, Other paralytic syndrome following unspecified cerebrovascular disease, bilateral, Other paralytic syndrome following unspecified cerebrovascular disease affecting unspecified side, Apraxia following unspecified cerebrovascular disease, Dysphagia following unspecified cerebrovascular disease , Facial weakness following unspecified cerebrovascular disease , Ataxia following unspecified cerebrovascular disease, Other sequelae following unspecified cerebrovascular disease , Intraoperative Cerebrovascular Infarction During cardiac surgery, Intraoperative cerebrovascular infarction during other surgery , Postprocedural cerebrovascular infarction during cardiac surgery, Postprocedural cerebrovascular infarction during other surgery, Personal history of transient ischemic attack (TIA), and cerebral infarction without residual deficits
CPT	61645, 37195	Intracranial mechanical thrombectomy and/or infusion for thrombolysis; thrombolysis, cerebral

**Supplemental Table 1D: Pulmonary Hypertension Hospitalization**		
**Code Type**	**Code**	**Description**
ICD-9	416.0, 416.8, 416.9	Primary pulmonary hypertension, other chronic pulmonary heart disease, chronic pulmonary heart disease unspecified

ICD-10	I27.0, I27.2, I27.8, I27.9	Primary pulmonary hypertension, other secondary pulmonary hypertension, other specified pulmonary heart disease, pulmonary heart disease unspecified
