# Supplementary material for: Identifying high risk clinical phenogroups of pulmonary hypertension through a clustering analysis
Source: PLoS One. 2023 Aug 25;18(8):e0290553. doi: 10.1371/journal.pone.0290553 (PMC10456132; doi:10.1371/journal.pone.0290553)
Supplement: S2 Table — (PDF) [file pone.0290553.s003.pdf]

S2 Table: Presumed WSPH group across clusters

Presumed WSPH Group	Cluster 1 (%)	Cluster 2 (%)	Cluster 3 (%)	Cluster 4 (%)	Cluster 5 (%)
Group 1	2.2	0.9	1.3	0.0	0.0
Group 2	27.3	36.5	23.1	64.4	81.5
Group 3	9.7	6.8	9.9	5.2	0.0
Group 4	2.2	0.7	2.3	0.3	0.0
Groups 1&2	0.7	0.6	0.6	0.6	0.8
Groups 1&3	0.6	0.3	0.3	0.0	0.0
Groups 1&4	0.1	0.0	0.2	0.0	0.0
Groups 2&3	4.9	3.7	6.9	15.3	14.6
Groups 2& 4	0.1	0.0	0.1	0.1	0.1
3 or more groups	52.1	50.7	55.4	14.1	3.0

WSPH groups defined as follows, group 1: patients with connective tissue disease; group 2: patients with left heart disease; group 3: patients with lung disease; group 4: patients with PE
