# Supplementary material for: Identifying high risk clinical phenogroups of pulmonary hypertension through a clustering analysis
Source: PLoS One. 2023 Aug 25;18(8):e0290553. doi: 10.1371/journal.pone.0290553 (PMC10456132; doi:10.1371/journal.pone.0290553)
Supplement: S3 Table — (PDF) [file pone.0290553.s004.pdf]

S3 Table. Clinical, hemodynamic and echocardiographic characteristics stratified by phenogroup for internal validation study

	Total (n=508)	Phenogroup 1 (n=73)	Phenogroup 2 (n=102)	Phenogroup 3 (n=121)	Phenogroup 4 (n=123)	Phenogroup 5 (n=89)	p value
**Demographics**							
Age, years†	64 (12)	44 (10)	69 (8)	68 (7)	66 (8)	69 (8)	<0.001
Male sex†	315 (62)	45 (62)	23 (23)	78 (64)	91 (74)	78 (88)	<0.001
BMI (kg/m^2)†	30.1 (6.9)	30.2 (7.2)	29.8 (6.6)	29.2 (6.7)	32.8 (7.3)	28 (5.4)	<0.001
**Clinical Comorbidities**							
Hypertension†	37 (62)	17 (23)	89 (87)	22 (18)	112 (91)	77 (87)	<0.001
Diabetes†	142 (28)	5 (7)	19 (19)	6 (5)	103 (84)	9 (10)	<0.001
Prior MI†	109 (21)	6 (8)	15 (15)	10 (8)	49 (40)	29 (33)	<0.001
Heart Failure†	206 (41)	15 (21)	21 (21)	11 (9)	87 (71)	72 (81)	<0.001
Valvular Disease†	187 (37)	13 (18)	73 (72)	13 (11)	22 (18)	66 (74)	<0.001
AF†	121 (24)	12 (16)	14 (14)	20 (17)	18 (15)	57 (64)	<0.001
OSA†	86 (17)	10 (14)	17 (17)	22 (18)	23 (19)	14 (16)	0.900
CLD†	103 (20)	9 (12)	22 (22)	15 (12)	35 (28)	22 (25)	<0.008
CKD†	15 (3)	0	1 (1)	1 (1)	8 (7)	5 (6)	0.010
**Laboratory**							
NT-pro BNP (pg/ml)	2179 [773-5203]	1127 [316-3800]	1637 [546-4272]	2243 [617-5215]	2573 [996-5805]	3595 [1567-6837]	0.002
**Echocardiography**							
LV EF (%)	50 (21)	52 (23)	59 (17)	56 (20)	42 (19)	44 (20)	<0.001
LV EDD (mm)	51 (10)	52 (11)	46 (7)	48 (10)	55 (10)	56 (9)	<0.001
RV systolic dysfunction (%)	104 (20)	15 (21)	8 (8)	32 (26)	24 (20)	25 (28)	0.003
MR (%)	147 (36)	18 (31)	30 (35)	28 (33)	28 (30)	43 (54)	0.008
TR (%)	108 (27)	12 (21)	14 (16)	25 (29)	24 (25)	33 (42)	0.003
**Hemodynamics**							
Mean PAP (mmHg)	30 [24-36]	30 [24-38]	26 [23-32]	30 [24-37]	30 [25-38]	31 [27-38]	<0.001
Mean RAP (mmgHg)	9 [6-12]	9 [6-12]	8 [6-11]	10 [6-12]	10 [7-13]	9 [6-13]	0.020
PCWP (mmHg)	18 [14-24]	18 [12-22]	18 [14-22]	16 [12-21]	21 [14-25]	20 [17-25]	<0.001
TPG (mmHg)	11 [8-16]	11 [8-16]	9 [6-13]	13 [8-20]	11 [8-15]	11 [8-15]	<0.001
PVR (WU)	2.2 [1.4-3.2]	2.1 [1.4-3.3]	2.0 [1.3-2.6]	2.6 [1.6-4.2]	2.2 [1.4-3.0]	2.4 [1.6-3.3]	0.010
PAPi	3.0 [2.2-4.4]	3.2 [2.0-4.6]	3.0 [2.2-4.7]	3.1 [2.0-4.4]	2.7 [1.8-3.9]	3.1 [2.0-4.6]	0.300
TD CI ($L/min/m^2$)	2.5 [2.1-2.9]	2.6 [2.1-3.5]	2.5 [2.2-2.9]	2.5 [2.0-2.9]	2.4 [2.1-3.0]	2.3 [1.9-2.5]	0.004
**PH Type**							
Pre-capillary PH	114 (22)	20 (27)	18 (18)	48 (40)	18 (15)	10 (11)	<0.001
Post-capillary PH	201 (40)	26 (36)	47 (46)	41 (34)	46 (37)	41 (46)	<0.001
Cpc-PH	129 (25)	16 (22)	20 (20)	21 (17)	40 (33)	32 (36)	<0.001
Other PH	64 (13)	11 (15)	17 (17)	11 (9)	19 (15)	6 (7)	<0.001

Data are expressed as number (%), mean (SD) or median [Q1-Q3]. Data provided is for MR and TR of mild to moderate severity and greater. Pre-capillary PH: PVR≥3WU, PCWP ≤15 mmHg; Post-capillary PH: PVR<3WU, PCWP>15 mmHg; Combined pre- and post-capillary PH (Cpc-PH): PVR≥3WU, PCWP>15 mmHg; Other PH: PVR<3WU, PCWP≤15 mmHg. AF, atrial fibrillation; OSA, obstructive sleep apnea; CLD: chronic lung disease; CKD, chronic kidney disease; LVEF, left ventricular ejection fraction; LVEDD, left ventricular end-diastolic dimension; MR, Mitral Regurgitation; TR, Tricuspid Regurgitation; PAP, pulmonary artery pressure; RAP, right atrial pressure; PCWP, pulmonary capillary wedge pressure; TPG, transpulmonary gradient; PAPi, pulmonary artery pulsatility index; TD, thermodilution; CI, cardiac index. †Denotes covariates used for clustering.
