# Supplementary material for: Identifying high risk clinical phenogroups of pulmonary hypertension through a clustering analysis
Source: PLoS One. 2023 Aug 25;18(8):e0290553. doi: 10.1371/journal.pone.0290553 (PMC10456132; doi:10.1371/journal.pone.0290553)
Supplement: S4 Table — (PDF) [file pone.0290553.s005.pdf]

S4 Table. Association between all-cause mortality, MACE events and PH admissions across phenogroups of internal validation study

Phenogroup	Events/n	HR	95% CI	P Value
	**ALL-CAUSE MORTALITY**			
**1**	20/73	reference		
**2**	35/102	0.63	0.31-1.28	0.2
**3**	44/121	0.81	0.42-1.56	0.52
**4**	68/123	1.22	0.66-2.26	0.52
**5**	48/89	1.13	0.57-2.23	0.74
	**MACE**			
**1**	22/71	reference		
**2**	34/98	1.65	0.84-3.24	0.15
**3**	33/117	1.31	0.67-2.58	0.43
**4**	68/121	2.86	1.57-5.21	< 0.001
**5**	41/88	2.63	1.34-5.17	0.005
	**PH ADMISSIONS**			
**1**	8/71	reference		
**2**	1/98	0.20	0.02-2.08	0.18
**3**	16/117	4.34	1.03-18.29	0.05
**4**	4/121	0.80	0.17-3.73	0.78
**5**	5/88	2.27	0.44-11.80	0.33

HR, hazard ratio; CI, confidence interval; MACE, major adverse cardiovascular events; PH, pulmonary hypertension
