# Supplementary material for: Identifying high risk clinical phenogroups of pulmonary hypertension through a clustering analysis
Source: PLoS One. 2023 Aug 25;18(8):e0290553. doi: 10.1371/journal.pone.0290553 (PMC10456132; doi:10.1371/journal.pone.0290553)
Supplement: S5 Table — (PDF) [file pone.0290553.s006.pdf]

S5 Table. **Post-hoc testing for between group differences in clinical characteristics**

Variable	Reference	Comparison p-value			
		**Cluster 2**	**Cluster 3**	**Cluster 4**	**Cluster 5**
**Demographics**					
Age	Cluster 1	<0.0001	<0.0001	<0.0001	<0.0001
Male sex	Cluster 1	<0.0001	0.8592	<0.0001	<0.0001
BMI	Cluster 1	0.0466	0.1384	<0.0001	<0.0001
**Clinical Comorbidities**					
Hypertension	Cluster 1	<0.0001	0.0393	<0.0001	<0.0001
Diabetes Mellitus	Cluster 1	<0.0001	<0.0001	<0.0001	<0.0001
Prior MI	Cluster 1	<0.0001	0.1141	<0.0001	<0.0001
Heart failure	Cluster 1	0.3050	<0.0001	<0.0001	<0.0001
Valvular disease	Cluster 1	<0.0001	<0.0001	0.8525	<0.0001
AF	Cluster 1	0.4955	<0.0001	0.1012	<0.0001
OSA	Cluster 1	0.0011	0.1952	0.0021	0.5274
Chronic lung disease	Cluster 1	<0.0001	0.0172	<0.0001	<0.0001
CKD	Cluster 1	0.2165	0.3366	<0.0001	0.0001
