# Supplementary material for: Identifying high risk clinical phenogroups of pulmonary hypertension through a clustering analysis
Source: PLoS One. 2023 Aug 25;18(8):e0290553. doi: 10.1371/journal.pone.0290553 (PMC10456132; doi:10.1371/journal.pone.0290553)
Supplement: S6 Table — (PDF) [file pone.0290553.s007.pdf]

S6 Table. Post-hoc testing for between group differences in laboratory, hemodynamic and echocardiographic characteristics

Variable	Reference	Comparison p-value			
		Cluster 2	Cluster 3	Cluster 4	Cluster 5
**Laboratory**					
NT-pro BNP (pg/ml)	Cluster 1	0.0554	0.5476	0.0173	<0.0001
**Hemodynamics**					
Mean PAP (mmHg)	Cluster 1	<0.0001	0.9275	0.9961	0.2230
Mean RAP (mmHg)	Cluster 1	<0.0001	0.0850	0.0327	0.4546
PCWP (mmHg)	Cluster 1	0.3528	0.014	<0.0001	<0.0001
TPG (mmHg)	Cluster 1	0.0022	0.0007	0.0577	0.0004
PVR (WU)	Cluster 1	0.9584	<0.0001	0.7467	0.7973
PAPi	Cluster 1	<0.0001	<0.0001	0.7012	0.0402
TD CI (L/min/m2)	Cluster 1	<.00001	<0.0001	<0.0001	<0.0001
Pre-capillary PH *	Cluster 1	<0.0001	0.0008	0.0006	<0.0001
Post-capillary PH *	Cluster 1				
Cpc-PH *	Cluster 1				
Other PH *	Cluster 1				
**Echocardiography**					
LVEF (%)	Cluster 1	<0.0001	<0.0001	<0.0001	0.0069
LVEDD (mm)	Cluster 1	<0.0001	0.0001	<0.0001	<0.0001
RV systolic dysfunction (%)	Cluster 1	0.5495	0.0001	0.8166	0.4516
MR (%)	Cluster 1	<0.0001	0.7625	0.7438	<0.0001
TR (%)	Cluster 1	0.9188	0.0006	0.6059	<0.0001

*Analysis performed using cumulative logit model
